# Review of catalytic reforming of biomass pyrolysis oil for hydrogen production

**DOI:** 10.3389/fchem.2022.962587

**Published:** 2022-07-26

**Authors:** Chengbing Zhang

**Affiliations:** Qingdao Cigarette Factory of Shandong China Tobacco Industry Co., Ltd., Qingdao, SD, China

**Keywords:** bio-oil, catalytic reforming, hydrogen, CO_2_ adsorption, biomass

## Abstract

After more than 20 years of development, biomass rapid pyrolysis technology has become technically mature. However, its main product biomass pyrolysis oil (bio-oil) has limited its application due to low energy density and poor thermal stability. Catalytic reforming is a workable way for bio-oil subsequent utilization to produce hydrogen. In this article, the research status and main research directions of bio-oil catalytic reforming for hydrogen production are summarized, with emphasis on CO_2_ adsorption-enhanced catalytic reforming for hydrogen production.

## Introduction

Hydrogen is one of the most desirable high calorific value clean energy sources, and catalytic reforming of hydrogen from bio-oil is an efficient way to produce hydrogen. In this article, we briefly introduce the recent progress of current biomass catalytic reforming hydrogen production technology in terms of mechanism, catalyst selection, new process, and new technology development. It also points out the current problems faced in this field, which can provide some new ideas and directions for future development.

The rapid pyrolysis of biomass to oil technology is gradually maturing, providing a new pathway for biomass to hydrogen. The US Renewable Energy Laboratory (Wang et al., 1998) pioneered the concept of a two-step hydrogen production process using rapid thermal cracking of biomass to prepare bio-oil followed by bio-oil reforming to produce hydrogen. The two-step biomass fast cracking-steam reforming approach to hydrogen production has the following advantages over other biomass to hydrogen technologies (An et al., 2008): 1) rapid biomass cracking technology has matured over the years and can be carried out at atmospheric pressure and is more economical; 2) the use of bio-oil is limited by its low energy density, however, bio-oil to hydrogen provides a subsequent processing route and an additional source of hydrogen production; 3) bio-oil is easier to transport than other solid biomass or hydrogen, and its processing plants for pyrolysis to oil and catalytic reforming to hydrogen can be flexibly located depending on the origin of the feedstock, local conditions, and scale of treatment.

### Catalytic reforming of bio-oil to hydrogen

The composition of bio-oil is more complex and is generally expressed as C_n_H_m_O_k_. Vapor is generally chosen as the gasification agent. Bio-oil and water vapor are both first reformed in the presence of a catalyst to produce CO and H_2_, and then CO and water vapor undergo a water-gas conversion reaction to produce CO_2_ and H_2_ as follows:

$${\text{C}}_{\text{n}}{\text{H}}_{\text{m}}{\text{O}}_{\text{k}}\;+\;\left(\text{n}-\text{k}\right){\text{ H}}_{2}\text{O }\to \text{ nCO }+\;\left(\text{n}+\text{m/2}-\text{k}\right){\text{ H}}_{\text{2 }}\left(\text{endothermic reaction}\right)$$ (1)

$$\text{nCO }+{\text{ nH}}_{\text{2 }}\text{O }\to {\text{ nCO}}_{2}{+\text{nH}}_{2}\;\left(\text{exothermic reaction}\right)\text{.}$$ (2)

The total reaction equation is expressed as:

$${\text{C}}_{\text{n}}{\text{H}}_{\text{m}}{\text{O}}_{\text{k }}{+\;\left(2\text{n}-\text{k}\right)\text{ H}}_{2}\text{O }\to {\text{ nCO}}_{2}\;+\;\left(\text{2n}+\text{m/2}-\text{k}\right){\text{ H}}_{\text{2 }}\text{.}$$ (3)

Since reaction temperatures are generally not lower than 500°C, thermal cracking reactions are unavoidable and disproportionation of CO may also occur.

$${\text{C}}_{\text{n}}{\text{H}}_{\text{m}}{\text{O}}_{\text{k}}\;\to {\text{ C}}_{\text{x}}{\text{H}}_{\text{y}}{\text{O}}_{\text{z}}\;+\text{ gases }({\text{H}}_{2}{\text{; H}}_{2}{\text{O; CO; CO}}_{2}{\text{; CH}}_{4}....)\;+\text{ coke}\text{.}$$ (4)

$$2\text{CO}\to {\text{CO}}_{2}\;+\text{ C}\text{.}$$ (5)

Current research on catalytic reforming of bio-oil to hydrogen is mostly at the laboratory stage. Since the composition of bio-oil is quite complex, with hundreds of oxygenated organic compounds, including ketones, ethanol (Haryanto et al., 2005), carboxylic acids (Wang et al., 1996), aldehydes, and phenols. The study of its mechanism is mostly limited to the study of single bio-oil model compounds, composite bio-oil model compounds, and bio-oil water soluble phases. Ethanol (Santamaria et al., 2020; Elharati et al., 2022), acetic acid (Bimbela et al., 2007; Vagia and Lemonidou, 2008; Choi et al., 2019), and toluene (Constantinou et al., 2010) are often used as single model compounds for the catalytic reforming of bio-oil to hydrogen or mixed in different ratios to make composite bio-oil model compounds (Chen et al., 2016).

Studies on the catalytic reforming of bio-oil molds have mostly focused on single components, mainly acetic acid, ethylene glycol, acetone, phenols, glucose, and glycerol, but there is a certain lack of reference for bio-oils with complex compositions. A mixture of methanol, ethanol, acetic acid, and acetone as light components and a mixture of furan, phenol, catechol, and m-methylphenol as heavy components were studied by the East China University of Science and Technology for their catalytic reforming to hydrogen, respectively (Wu et al., 2008b; Xu et al., 2010). Experiments have shown that lighter fractions can achieve higher hydrogen yields and carbon conversions at lower temperatures and water-to-carbon ratios, while heavy fractions require more demanding conditions.

Marquevich et al. (1999) investigated the catalytic reforming of acetic acid, cresol, benzyl ether, glucose, xylose, and sucrose to hydrogen using a packed bed reactor. When the reaction temperature was higher than 650 °C, the first three substances were completely converted to H_2_ and CO_2_, but when the temperature was lower than 650 °C, the hydrogen yield decreased to 70–90% due to the thermal cracking reaction; however, the three sugars were more susceptible to thermal cracking, which occurred before they came into contact with the catalyst to produce coke. Wang et al. (2007) investigated the catalytic reforming of bio-oil to hydrogen using a homemade C1_2_A_7_/MgO catalyst on a packed bed reactor and found that the addition of MgO effectively suppressed carbon build-up and hydrogen yield could reach 82%, but carbon deposition still occurred after a period of time, which led to a rapid decrease in hydrogen yield.

## Progress in catalytic reforming of bio-oil for hydrogen production research

### Development of catalysts with high activity and high stability

Nickel-based catalyst is a kind of catalyst which has been widely studied in the steam catalytic reforming of bio-oil. Nickel has high activity for C-C (carbon atoms linked by a bond) breaking, high selectivity for H_2_ generation, and relatively low price. However, the catalytic activity of ordinary nickel catalyst is limited, and it is easy to passivate in the reforming process, so it usually needs to be modified. Yan et al. (2010a) found that the catalytic performance of nickel-based catalysts with CeO_2_ as an additive and ZrO_2_ as a carrier for bio-oil reforming was significantly higher than that of commercial nickel catalysts. Iriondo et al. (2008) compared the catalytic performance of nickel-based catalysts with different elements (Mg, Zr, Ce, and La) for glycerol reforming. It was found that the catalyst with Zr had outstanding selectivity and its gas composition was similar to the thermodynamic prediction.

**FIGURE 1:**
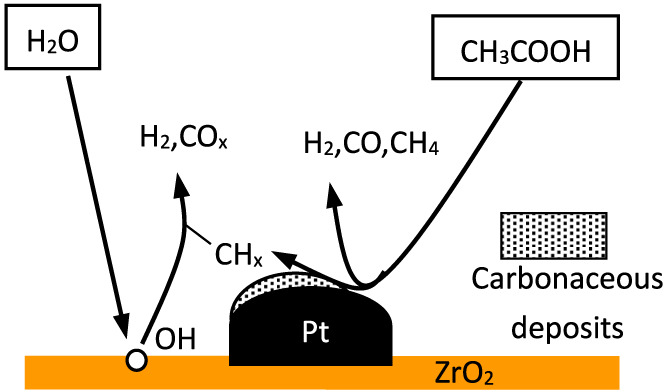
Schematic of bifunctional mechanism of Pt-ZrO_2_ catalytic reforming of acetic acid for hydrogen production.

Takanabe et al. (2004) studied acetic acid reforming reaction with Pt-ZrO_2_ as a catalyst and found that acetic acid was almost fully converted. Tanabe described the bifunctional mechanism of Pt-ZrO_2_ catalytic steam reforming of acetic acid for hydrogen production as shown in Figure 1. Pt causes acetic acid to produce H_2_, CO_x_, CH_4_, and carbon residue mainly in the form of CH_x_, which tends to block the Pt surface. ZrO_2_ can activate H_2_O, and the generated hydroxyl groups can gasify CH_x_ on the surface of Pt, producing H_2_ and CO_2_, so that the catalyst can be recycled.

Natural ores such as calcite (Papadopoulou and Ioannides, 2020), dolomite (Valle et al., 2020), olivine (Acha et al., 2020), and so on (Liu et al., 2020) are also often used as carriers for catalytic reforming of bio-oils for hydrogen production because of their low cost, wide distribution, abundance of species, and relatively high catalytic efficiency. Studies have shown that the optimal conversion of bio-oil by Fe-olivine is 97.2% (Quan et al., 2017). Five different sources of calcite for the reforming of phenol were calcinated at 850 °C, and the results showed that the conversion of phenol was slightly higher than 30% at 0.3 g catalyst, 1.2 ml/min feed, and 700 °C (Constantinou et al., 2010).

### Development of new reactor

At present, the catalytic reforming of bio-oil to hydrogen production is mostly carried out in packed bed reactors, which can easily lead to carbon deposition on the surface of the catalyst and the free space of the reactor. Therefore, it is necessary to develop a new and efficient bio-oil reforming hydrogen reactor.

Wu et al. (2008a) established a catalytic reforming system for a two-stage fixed bed reactor of bio-oil. In the first stage, cheap dolomite was filled as a catalyst, and the bio-oil was preliminarily steam reformed so as to prevent the bio-oil from directly contacting the metal catalyst in the second stage reactor and slow down its passivation. In the second stage, Ni/MgO was filled to further catalytic reforming of primary products in the first stage. The experiment showed that the potential hydrogen yield in the first-stage reforming gas production is obviously improved, the methane can be completely converted in the second-stage reforming process, and the ideal gas concentration can reach 100%.

**FIGURE 2:**
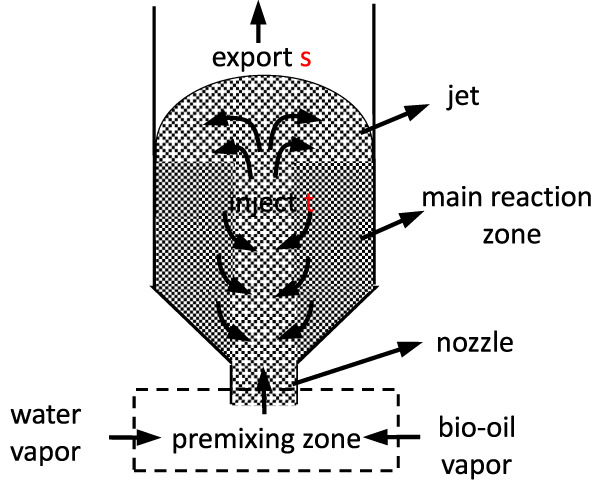
Schematic of spouted bed reactor.

Kechagiopoulos et al. (2007; 2009) developed a spouted bed reactor as shown in Figure 2. After being premixed and heated, water vapor and bio-oil vapor enter the reactor from the bottom nozzle, carrying catalyst particles to form a jet in the central area of the reactor, thus realizing dilute phase transmission of catalytic reaction, while the surrounding dense phase moves slowly through the mutual exchange of gas and particles. When the jet momentum is high enough, the jet stream is sprayed into the upward part of the bed, where the catalyst particles carried by the gas are separated from the produced gas, forming an arc-shaped streamline that falls back into the bed. Using this reactor, the authors studied ethylene glycol and found that the coking phenomenon was completely avoided. Nickel/olivine shows excellent catalytic properties because of its high mechanical strength and coking resistance. The yield of hydrogen can reach more than 80%.

Although there are many aspects of the new reactors that are advantageous, there are also some disadvantages. First, the poor catalyst mobility in the bio-oil two-stage fixed bed reactor will lead to coke formation and thus catalyst deactivation (Ochoa et al., 2020; Landa et al., 2022; Zhang et al., 2022). Second, the spouted bed reactor imposes higher requirements on the operating conditions. For example, the bio-oil cannot be completely gasified during premixing, and some of the solid residues produced will block the nozzles and thus affect the premixing effect. Also, as with fluidized bed reactors (Medrano et al., 2009; Arandia et al., 2017), there is a disadvantage of catalyst wear, so a high degree of catalyst hardness is required.

### Development of new technology

University of Science and Technology of China (Chen et al., 2009; Ye et al., 2009; Lin et al., 2010) proposed the electro-catalytic reforming process for hydrogen production from bio-oil. It was found that compared with the conventional reforming process, the electrocatalysis greatly promoted the bio-oil reforming reaction, and the hydrogen yield and carbon conversion were significantly improved. The hydrogen yield could reach 93.5% at 400°C. The author holds that electrons will burst out from the solid surface when the charged metal or metal oxide is heated to a certain temperature. These thermal electrons can effectively reduce the metal oxidation state of the catalyst to the metal state, which leads to the enhancement of the catalyst activity. At the same time, the thermal electrons can decompose and reform the organic matter, resulting in the formation of some unstable molecular fragments and high reaction activities of free radicals and thus improving the reform effect.

Xie et al. (2014) and Xie et al. (2015b) carried out the thermodynamic analysis and experimental research on steam reforming of bio-oil. It was found that the hydrogen concentration was basically stable at about 70% and the CO_2_ concentration was more than 20%, which limited the application of this gas production. As can be seen from the total reaction equation of hydrogen production from bio-oil steam reforming, when CO_2_ concentration is reduced, the reaction will proceed in a direction conducive to hydrogen production, while inhibiting the formation of carbon deposition to some extent. In addition, by adsorbing CO_2_ in the gas production, the content of CO, CO_2_, and CH_x_ in the gas production can be reduced so as to improve the purity of hydrogen in the gas production. Therefore, the CO_2_ adsorption-enhanced catalytic reforming process for hydrogen production was proposed.

Chemical cycling is also known as a promising method for H_2_ production from solid fuels (Situmorang et al., 2020). This method can effectively use carbonaceous materials as reducing agents, redox rings of metal oxides as oxygen carriers, and steam as an oxidizer and hydrogen source. In particular, it can produce pure H_2_ and other gases separately by coupling several reactors without using any additional gas treatment and separation processes (Zhang et al., 2020). Combining the steam bio-oil reforming process with the chemical looping unit using biochar for H_2_ production can increase H_2_ production efficiency from biomass by more than 50% (Situmorang et al., 2020).

## CO_2_ adsorption-enhanced catalytic reforming for hydrogen production

Yan et al. (2010b) and Dou et al. (2010) used calcined dolomite as a CO_2_ adsorbent to study the catalytic reforming of CO_2_ adsorption in glycerol and bio-oil aqueous phase to produce hydrogen in a packed bed reactor. They found that the yield and concentration of hydrogen were significantly improved compared with those without adsorbents, and the hydrogen concentration could reach about 90% or even higher. However, as time goes on, the adsorption capacity of the adsorbent for CO_2_ will tend to be saturated, and the concentration of CO_2_ in gas production will gradually increase, thereby inhibiting the total reaction from proceeding in the positive direction, and the hydrogen yield will decrease.

**FIGURE 3:**
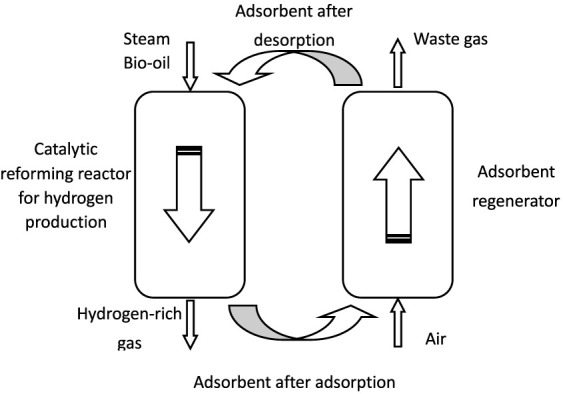
Conceptual diagram of the continuous adsorption-enhanced reforming to hydrogen process.

Therefore, Chen et al. (2011), Xie et al. (2015a), and Xie et al. (2016) proposed the idea of setting up a regeneration reactor outside the reforming reactor to achieve continuous and efficient hydrogen production while achieving adsorbent recycling as shown in Figure 3. The bio-oil and water vapor were mixed and entered into the reformer, while the adsorbent was carried into the reformer by gas. The steam catalytic reforming reaction is carried out in the reformer, and the adsorbent adsorbs the CO_2_ produced by the reforming reaction; gas-solid separation was carried out for gas-producing and unreacted water vapor as well as adsorbent (after adsorptions), hydrogen-rich gas was collected after purification, and adsorbent (after adsorption) was separated and entered into the regenerator. The adsorbent (after adsorption) desorbed CO_2_ in the regenerator was regenerated and entered into the reforming reactor for recycling. Theoretically, the process can obtain high hydrogen yield and purity and realize the continuous operation of the system. However, the process still faces the following key problems: in order to ensure the smooth passage of the adsorbent through the catalyst bed, the catalyst bed structure should have both rich and large voids, and the adsorbent should have enough small particle size; in order to ensure that the adsorbent has sufficient residence time in the catalyst bed, the direct channel bed structure is not acceptable (such as the honeycomb structure). Therefore, as two core components in the continuous CO_2_ adsorption-enhanced bio-oil catalytic reforming process, the adsorbent should be micro-powder, and the catalyst should be large particles with strong compressive and wear resistance.

Xie et al. (2016) prepared granular Ce-Ni/Co catalysts and carried out an experimental study on the adsorption-enhanced bio-oil reforming for hydrogen production with powdered CaO as a CO_2_ adsorbent, and the study found that after the addition of adsorbent, the hydrogen concentration could be stabilized at more than 90%, the hydrogen yield was greatly improved, and the carbon coking on the catalyst surface was also significantly inhibited. However, the flow, mass transfer, and heat transfer in the reforming process were not involved, and the related research on the other core component, the adsorbent regenerator, and the whole process system was not carried out.

The most commonly used adsorbents are calcium-based adsorbents. However, when CaO is used as an adsorbent, the CO_2_ adsorption capacity decreases significantly after repeated cyclic adsorption-regeneration, mainly because the high-temperature environment makes CaO agglomerate and the porous channel blocked, which hinders the internal diffusion of CO_2_. At the same time, the repeated carbonation reactions of CaO and CO_2_ also change the pore structure. Martavaltzi and Lemonidou (2008) improved the adsorbent, and the CaO-Ca_12_Al_14_O_33_ adsorbent was prepared by adding Al(NO_3_)_3_.9H_2_O into CaO. The experimental results show that the adsorbent has high adsorption performance and can maintain high adsorption capacity for a long time.

## Conclusion

The production of hydrogen *via* catalytic reforming of pyrolysis bio-oil not only provides a follow-up utilization way for the rapid pyrolysis products but also increases the source of hydrogen energy. How to improve the reaction index and reduce carbon deposition is the core issue of bio-oil catalytic reforming for hydrogen production. The current research mainly focuses on the manufacture of catalysts, the optimization of reactors, and the development of new processes.

1) Super performance catalyst can promote the bio-oil reforming reaction, and in the meantime, it has high selectivity to hydrogen and long service life. The present research mainly focuses on nickel-based catalysts. Although some achievements have been made, exploring catalysts with higher activity and longer life is still one of the main directions for hydrogen production from bio-oil reforming.

2) At present, the research on the bio-oil catalytic reforming reactor is mostly concentrated on a packed bed reactor, which is prone to catalyst passivation and reactor blockage. In order to reduce catalyst passivation and make the reforming hydrogen production reaction more fully, it is particularly important to develop new reactors.

3) Adsorption-enhanced reforming process can obtain higher hydrogen yield and concentration. At present, the adsorbents used for bio-oil adsorption-enhanced reforming for hydrogen production are mainly calcium-based adsorbents. Such adsorbents are prone to sintering agglomeration under a high-temperature environment, which affects the adsorption performance. Therefore, it is particularly important to explore new CO_2_ adsorbents.
